# Glucose-6-phosphate dehydrogenase deficiency and the use of primaquine: top-down and bottom-up estimation of professional costs

**DOI:** 10.11606/S1518-8787.2017051007084

**Published:** 2017-11-07

**Authors:** Henry Maia Peixoto, Marcelo Augusto Mota Brito, Gustavo Adolfo Sierra Romero, Wuelton Marcelo Monteiro, Marcus Vinícius Guimarães de Lacerda, Maria Regina Fernandes de Oliveira

**Affiliations:** I Núcleo de Medicina Tropical. Universidade de Brasília. Brasília, DF, Brasil; II Instituto de Avaliação de Tecnologia em Saúde. Porto Alegre, RS, Brasil; III Fundação de Medicina Tropical Dr. Heitor Vieira Dourado. Manaus, AM, Brasil; IV Universidade Estadual do Amazonas. Manaus, AM, Brasil; V Instituto Leônidas e Maria Deane. Fundação Oswaldo Cruz. Manaus, AM, Brasil

**Keywords:** Glucose-6-phosphate dehydrogenase deficiency, economics, Primaquine, adverse effects, Malaria, prevention & control, Health Care Costs, Unified Health System, economics, Deficiência de glicose-6-fosfato desidrogenase, economia, Primaquina, efeitos adversos, Malária, prevenção & controle, Custos de Cuidados de Saúde, Sistema Único de Saúde, economia

## Abstract

The aim of this study has been to study whether the top-down method, based on the average value identified in the Brazilian Hospitalization System (SIH/SUS), is a good estimator of the cost of health professionals per patient, using the bottom-up method for comparison. The study has been developed from the context of hospital care offered to the patient carrier of glucose-6-phosphate dehydrogenase (G6PD) deficiency with severe adverse effect because of the use of primaquine, in the Brazilian Amazon. The top-down method based on the spending with SIH/SUS professional services, as a proxy for this cost, corresponded to R$60.71, and the bottom-up, based on the salaries of the physician (R$30.43), nurse (R$16.33), and nursing technician (R$5.93), estimated a total cost of R$52.68. The difference was only R$8.03, which shows that the amounts paid by the Hospital Inpatient Authorization (AIH) are estimates close to those obtained by the bottom-up technique for the professionals directly involved in the care.

## RESUMO

A pesquisa teve por objetivo estudar se o macrocusteio, baseado no valor médio identificado no Sistema de Internação Hospitalar (SIH/SUS), constitui um bom estimador do custo de profissionais de saúde por paciente, tendo como comparação o método de microcusteio. O estudo foi desenvolvido no contexto da assistência hospitalar oferecida ao portador da deficiência de glicose-6-fosfato desidrogenase (dG6PD) do sexo masculino com evento adverso grave devido ao uso da primaquina, na Amazônia Brasileira.

O macrocusteio baseado no gasto em serviços profissionais do SIH/SUS, como proxy desse custo, correspondeu a R$60,71, e o microcusteio, baseado nos salários do médico (R$30,43), do enfermeiro (R$16,33) e do técnico de enfermagem (R$5,93), estimou um custo total de R$52,68. A diferença foi de apenas R$8,03, mostrando que os valores pagos pela Autorização de Internação Hospitalar (AIH) são estimadores próximos daqueles obtidos por técnica de microcusteio para os profissionais envolvidos diretamente no cuidado.

## INTRODUCTION

Primaquine, an 8-aminoquinoline, is currently the only drug licensed in Brazil for the radical treatment of *Plasmodium vivax* malaria. It contributes to avoid relapses in this type of malaria and, consequently, decreases the number of persons with this parasite. However, this medication has been associated with severe hemolysis, hospitalization, and even death of male individuals with glucose-6-phosphate dehydrogenase (G6PD) deficiency, being the main threat to persons with G6PD deficiency in Brazil^1^ ^,^ ^2^. In the Brazilian Amazon, the estimated prevalence of G6PD deficiency is 4.5%^3^. Estimates presented by Peixoto et al.^2^ indicate that G6PD deficiency in the field of malaria is costly for the Brazilian Unified Health System (SUS), especially from the hospitalization costs of patients with severe adverse events associated with the use of primaquine. In this context, the cost-effectiveness analysis presented by Peixoto et al.^4^ shows that the use of the CareStart^TM^ G6PD rapid diagnostic test before the administration of primaquine is efficient to diagnose G6PD deficiency and to avoid hospitalization in the Brazilian Amazon.

The validity of economic evaluations depends, largely, on the method and accuracy of the costs evaluated^5^. Costs can be obtained from the detailing of items and resources used (bottom-up), from global estimates (top-down), or from mixed approaches that use both bottom-up and top-down. An approach based on top-down may be more adequate when the variability of a given region needs to be represented, increasing the external validity of the evaluation; on the other hand, bottom-up reflects the characteristics of a sample, which limits the generalization, despite being more precise^6^.

In Brazil, the Hospitalization System (SIH/SUS) provides the values of hospitalizations paid by the SUS to health institutions, which subsidizes the overall costs according to the cause of hospitalization and contemplates hospital and professional services. Because of the debate on the underfunding related to the amounts paid by the SUS, the development of bottom-up methodologies in order to estimate hospital costs could contribute to more accurate economic analyses.

The purpose of this study was to identify whether the top-down method for professional services, based on the average value paid for these services identified in the SIH/SUS, is a good estimator of the cost of health professionals. This was carried out in relation to the bottom-up method based on the salaries of the physician, nurse, and nursing technician in the context of hospital care given to the male patient with G6PD deficiency with severe adverse event from the use of primaquine in the Brazilian Amazon.

## METHODS

A comparison of costing methods was carried out in the context of an economic analysis developed from the perspective of the SUS for 2013, considering all nine States of the Brazilian Amazon. We analyzed the cost of professional service with the hospitalization of patients with G6PD deficiency submitted to the radical treatment of *P. vivax* infection with primaquine. To obtain this cost, we used two techniques: the top-down, performed from an administrative database based on the Professional Service component of the Hospitalization Authorization (AIH) paid by SUS, and the bottom-up, based on the casuistry of the Fundação de Medicina Tropical Dr. Heitor Vieira Dourado (FMT-HVD), a center of reference in malaria care and research in the State of Amazonas, fully funded by the SUS, located in the city of Manaus.

The top-down technique obtained the average value of the Professional Service component of the AIH from the analysis of the SIH/SUS databases, using the following procedure: the 108 files that contain the SIH/SUS registry data from the nine States of the Brazilian Amazon, of 2013, were obtained in the *.dbf format and exported to the Statistical Package for the Social Sciences (SPSS®) software, version 20.0, being transformed into the *.sav format; the databases were analyzed individually in the SPSS®. Using the localization function of the SPSS®, we identified the male individuals older than six months hospitalized for the treatment of hemolytic anemia from G6PD deficiency (ICD-10 D550). Subsequently, the results of the analyses were grouped into a single file, allowing us to obtain the average value of said component. Although the professional service component of the AIH refers mainly to the costs of the physician’s service, this component was also used as a proxy for the total professional costs (physician, nurse, and nursing technician), since we cannot discriminate the cost of the service of other professionals from the hospital service component of the AIH.

The bottom-up technique was based on the average salaries practiced by the State of Amazonas in 2013, for the physician, nurse, and nursing technician (total professional cost), which allowed us to compare the total professional cost, physician cost, and the value obtained from the analysis of the professional service component of the AIH. From the monthly salary, we identified the value of one hour of work (monthly salary divided by the monthly work hours), followed by its multiplication by the estimated hours dedicated to the patient, which was obtained by multiplying the average number of days of hospitalization (4.58 days) and 24 hours. This product was then weighted by the proportion of patients hospitalized for G6PD deficiency at the FMT-HVD, since health professionals do not exclusively treat patients hospitalized for G6PD deficiency (Table). The average number of days hospitalized and the number of patients hospitalized for G6PD deficiency who subsidized the calculation of the proportion of inpatients were obtained from unpublished information from the monitoring of 33 males with G6PD deficiency, and mean age of 24.3 years, hospitalized at FMT-HVD between 2009 and 2011 for the adverse events of primaquine. Thus, the proportion of inpatients with G6PD deficiency was calculated based on the division between the 33 hospitalizations of patients with enzyme deficiency and the 8,654 hospitalizations from all causes in the institution in the same period (amounting to 0.38%).

**Table:** Estimation of the cost of professional services involved in the hospitalization of a patient carrier of G6PD deficiency infected by P. vivax after the use of primaquine in the Brazilian Amazon, 2013.

Professionals	(A)	(B)	(C)	(D)	(E1)	Análise de sensibilidade	
					
	Monthly Salary (R$)	Work hours per week	Work hours per month	Value of the hours worked	Professional cost per patient	(E2)	(E3)
				
			C = B*4	(A/C)	E = D*4.58^a^*24h*0.38%^b^	Proportion of inpatients (0.25%–0.51%)^c^	Average days of hospitalization (2.58–6.57)^d^
Physician	5,832.98	20	80	72.91	30.43	20.02–40.84	17.16–43.69
Nurse	4,694.41	30	120	39.12	16.33	10.74–21.91	9.20–23.44
Nursing technician	1,704.1	30	120	14.21	5.93	3.90–7.95	3.34–8.51
Total					52.68	34.66–70.70	29.70–75.64

Difference between costs: top-down versus bottom-up				Difference 1^e^	30.28	19.87–40.69	17.02–43.55
				Difference 2^f^	8.03	-9.99–26.05	-14.93–31.01

^a^ Average days of hospitalization for G6PD deficiency at FMT-HVD.

^b^ Proportion of hospitalization for G6PD deficiency among the inpatients at FMT-HVD.

^c^ Variation of the professional cost per patient based on the confidence interval (95%CI) of the proportion of inpatients for G6PD deficiency among the inpatients at FMT-HVD.

^d^ Variation of the professional cost per patient based on the confidence interval (95%CI) of the average days of hospitalization for G6PD deficiency at FMT-HVD. ^e^ Difference between top-down (R$60.71) and bottom-up based only on the physician cost.

^f^ Difference between top-down (R$60.71) and bottom-up based on the total professional cost (physician, nurse, and nursing technician).

Univariate sensitivity analyses were performed based on the 95% confidence intervals (95%CI) of the following parameters: proportion of hospitalization from G6PD deficiency and average days of hospitalization.

## RESULTS

The average cost of professional services obtained by the top-down technique, based on the analysis of hospitalizations identified in the SIH/SUS, corresponded to R$60.71. The bottom-up, based on the salaries of the physician, nurse, and nursing technician, indicated a cost of R$52.68, with a preponderance of the cost with the physician (R$30.43), followed by the nurse (R$16.33), and the nursing technician (R$5.93). We can observe that the difference between the costs obtained from the different techniques, considering the total professional cost, was only R$8.03, and considering only the physician costs it was R$30.28 (Table). The univariate sensitivity analysis performed with the proportion of hospitalization and the average number of days hospitalized for G6PD deficiency showed that the total professional cost obtained using the bottom-up method ranged between R$34.66 and R$70.70 and between R$29.70 and R$75.64 (Table), respectively.

## DISCUSSION

The hospital costs obtained from the analysis of the regional and national registries of the SIH/SUS have subsidized several economic analyses developed from the SUS perspective, thus enabling, according to Silva et al.^7^, a more comprehensive view. According to the guidelines for the economic evaluation proposed by the Ministry of Health (MS), the hospitalization costs obtained from the AIH values have been used in almost all studies conducted in Brazil, although the AIH is criticized for representing the values reimbursed and not necessarily the actual costs^6^. In this context, we can observe the use of the average value of the AIH, which was obtained from the analysis of the SIH/SUS, in studies with the most varied objectives, such as: disease cost assessment^2^ ^,^ ^8^, analysis of vaccine efficacy^9^ ^,^ ^10^, efficiency of new diagnostic technologies^4^, efficiency of programs^11^, and efficiency of new therapeutic technologies^12^, among others.

The discussion about the devaluation of the amounts paid by the AIH should also be considered in economic analyses. In this regard, Sampaio et al.^13^, when studying the financial management of neurosurgery, have compared the amounts paid by the AIH with the expenses cataloged by patients during the period from August 2012 to June 2013. The authors^13^ have identified a deficit of R$395,329.17 between the amounts obtained by the bottom-up technique (R$718,036.70) and the amount reimbursed by the AIH (R$321,607.45). Lucarevschi et al.^14^, when combining the bottom-up and top-down techniques to assess direct hospital costs related to pneumococcal meningitis in children, have identified costs 10 to 20 times higher than those paid by the AIH. Given the variety of health outcomes, we can assume that the amount paid by the AIH may also overestimate the cost of hospitalization for some outcomes, although this study has not identified publications that support this assumption.

In this study, the bottom-up value of professional services, initially considering only the cost of the physician, compared to the top-down, presented value corresponding to half of the amount reimbursed by the AIH. However, when simultaneously considered the costs of the physician, the nurse, and the nursing technician, the value obtained was very close to the top-down value, indicating that the AIH can be a good proxy of the estimation of costs of services of the professionals directly involved in the care of persons with G6PD deficiency when compared with the bottom-up technique.

This study presents some limitations, such as the fact that some epidemiological parameters and the costs used in the bottom-up technique come from the casuistry of the State of Amazonas and they are extrapolated to the entire Brazilian Amazon. The uncertainties considered in the univariate sensitivity analysis indicated that the professional costs did not show significant variations, although they were influenced by the variation of the proportion of hospitalized patients for G6PD deficiency and days of hospitalization.

The comparison between the bottom-up and top-down methods is an unprecedented contribution to the discussion of costing techniques for professional costs in malaria care settings in the Brazilian Amazon, with the potential to support future economic studies in the area. It should be noted that the AIH is widely used in Brazil as a source of hospital care costs, as it facilitates the comparison between economic studies and represents the values practiced in the service routine.
